# Islands beneath islands: phylogeography of a groundwater amphipod crustacean in the Balearic archipelago

**DOI:** 10.1186/1471-2148-11-221

**Published:** 2011-07-26

**Authors:** Maria M Bauzà-Ribot, Damià Jaume, Joan J Fornós, Carlos Juan, Joan Pons

**Affiliations:** 1 Departament de Biologia, Universitat de les Illes Balears, Edifici Guillem Colom, Campus Universitari, ctra. Valldemossa, km 7.5, 07122-Palma de Mallorca, Balearic Islands, Spain; 2 IMEDEA (CSIC-UIB), Instituto Mediterráneo de Estudios Avanzados, c/Miquel Marquès, 21, 07190-Esporles, Balearic Islands, Spain; 3 Karst and Littoral Geomorphology Research Group, Universitat de les Illes Balears, Edifici Guillem Colom, Campus Universitari, ctra. Valldemossa, km 7.5, 07122-Palma de Mallorca, Balearic Islands, Spain

## Abstract

**Background:**

Metacrangonyctidae (Amphipoda, Crustacea) is an enigmatic continental subterranean water family of marine origin (thalassoid). One of the species in the genus, *Metacrangonyx longipes*, is endemic to the Balearic islands of Mallorca and Menorca (W Mediterranean). It has been suggested that the origin and distribution of thalassoid crustaceans could be explained by one of two alternative hypotheses: (1) active colonization of inland freshwater aquifers by a marine ancestor, followed by an adaptative shift; or (2) passive colonization by stranding of ancestral marine populations in coastal aquifers during marine regressions. A comparison of phylogenies, phylogeographic patterns and age estimations of clades should discriminate in favour of one of these two proposals.

**Results:**

Phylogenetic relationships within *M. longipes *based on three mitochondrial DNA (mtDNA) and one nuclear marker revealed five genetically divergent and geographically structured clades. Analyses of cytochrome oxidase subunit 1 (*cox1*) mtDNA data showed the occurrence of a high geographic population subdivision in both islands, with current gene flow occurring exclusively between sites located in close proximity. Molecular-clock estimations dated the origin of *M. longipes *previous to about 6 Ma, whereas major cladogenetic events within the species took place between 4.2 and 2.0 Ma.

**Conclusions:**

*M. longipes *displayed a surprisingly old and highly fragmented population structure, with major episodes of cladogenesis within the species roughly correlating with some of the major marine transgression-regression episodes that affected the region during the last 6 Ma. Eustatic changes (vicariant events) -not active range expansion of marine littoral ancestors colonizing desalinated habitats-explain the phylogeographic pattern observed in *M. longipes*.

## Background

Subterranean fauna provides unique opportunities for the study of evolutionary mechanisms and speciation processes [1]. In recent years, phylogeographic analyses have revealed unprecedented cases of cryptic speciation, restricted distribution and presumed sympatric speciation among different cave-dwelling animal groups [2]. Nevertheless, the occurrence of extensive morphological conservatism in subterranean fauna frequently hampers the establishment of phylogenetic inferences based solely on morphological features. In this context, homoplasy arises from common exposure to the particular selective pressures inherent to cave life (i.e., darkness and oligotrophy) or from the lack of directional selection [3,4]. Conversely, isolation in caves can lead these morphologically undifferentiated subterranean organisms to display high levels of genetic divergence [4-6].

Geological and hydrological processes, in particular shifts in water tables, can lead to the isolation or connection of aquifers, with consequent effects on gene flow between populations of subterranean aquatic organisms [6]. In the same way, marine regressions are suggested to have played a major role in the isolation of many marine relicts in continental groundwaters [7-10]. Recent molecular phylogenetic and phylogeographic studies on subterranean amphipods emphasize the role played by historical factors (i.e., glacial or drought episodes) in the pattern of genetic diversification and distribution displayed by these animals [5,6,11]. Likewise, [12] considered the influence of contingency, i.e., whether the colonization event involved a single localized surface ancestor or multiple, geographically separated ancestors, on the shaping of these patterns. In addition, larval life history traits, such as feeding mode (planktotrophic vs. lecithotrophic) can play a determinant role in crustacean distribution, as they control the duration of the dispersive phase [13,14]. However, stygobiont amphipods have a comparatively reduced dispersal potential (as do all peracarid crustaceans), as the females carry offspring in a marsupium and these are brooded and not released into the water column until metamorphosed into diminutive non-natatory adults [15].

Among the obligate dwellers of subterranean waters (stygobionts), a high number belong to so-called thalassoid lineages, organisms that are derived directly from marine ancestors [7]. Thalassoid forms are known to occur among a vast array of faunistic groups, especially the Crustacea [16,8]. The ancestors of thalassoid animals presumably inhabited marine transitional habitats, such as submarine fissures, mixohaline submarine karstic springs or the interstitial medium developed in sandy and gravelly coastal sediments, where sharp variations in salinity (i.e., periodical exposure to desalinated waters) and other environmental conditions mimic, in some way, those found in fresh groundwaters [7]. Colonization of inland freshwater aquifers by this preadapted marine fauna might have proceeded as a natural extension of their primary niche, followed by an adaptive shift; this process would be independent of the occurrence of environmental constraints, such as episodes of glaciation, drought or marine regression [17,18]. This hypothesis provides a plausible explanation for the origin of some freshwater stygobiont ostracods closely related to marine euryhaline taxa [19]. However, most faunistic and biogeographic evidence favours an alternative vicariant scenario by which colonization occurs passively via stranding of ancestral populations during episodes of marine regression [7-10]. Accordingly, sea withdrawal or tectonic uplift at different geological periods could have led to the gradual isolation of populations of ancestral marine taxa in inland groundwaters, triggering their ulterior diversification and speciation. This hypothesis explains satisfactorily the distribution of many stygobiont crustaceans and is testable by collating a phylogenetic framework and molecular-clock-age estimates of relevant clades, with their respective geographic distributions [2,20].

Here, we studied the phylogeography of *Metacrangonyx longipes *Chevreux, 1909, a euryhaline stygobiont amphipod crustacean that is endemic to Mallorca and Menorca (Balearic Islands; W Mediterranean). On Mallorca, it occurs in various types of groundwater habitats, from coastal anchialine caves (*sensu *[21]) of raised salinity to freshwater inland wells, caves and springs. On Menorca, the species is restricted to coastal anchialine caves and wells and is absent from fresh inland groundwaters. On both islands, the species is limited to lowlands and is absent in apparently suitable habitats located at elevations higher than 125 m above sea level. The Metacrangonyctidae is a strictly inland water subterranean family with no close relatives; however, several lines of evidence strongly suggest its marine origin: (1) its members are known only from continental regions that were covered by ancient epicontinental seas [22,23]; and (2) several species still maintain ties with the marine environment (i.e., they live in anchialine wells and caves in coastal areas; [23]).

In this study, we used the sequences of three mitochondrial and one nuclear gene of *M. longipes *and of several congeneric species to perform a phylogenetic analysis of the species and infer population divergence times. Moreover, we use sequences of the cytochrome oxidase subunit 1 gene from a more comprehensive data set to perform a phylogeographic analysis and to examine the population structure of this taxon. Given the manifested euryhalinity of *M. longipes *and the absence of any appreciable morphological differentiation between its populations on the two islands, our initial prediction was that the species could have dispersed across the groundwater environment of the islands using the virtually continuous peripheral coastal anchialine pathway, from which it could have colonized inland freshwater habitats recurrently. If this was the case, we could expect a pattern of considerable gene flow and shallow genetic divergences within each island, with genetic signatures of inland populations deriving from coastal ones. However, our study revealed that this amphipod displays a remarkably ancient and highly fragmented population structure, with episodes of cladogenesis that could be related to major sea-level changes that affected the islands during the last 6 Ma.

## Results

Four gene fragments-three mitochondrial (cytochrome oxidase subunit 1 (*cox1*), cytochrome b (*cob*) and 16S rRNA (*rrnL*)) and one nuclear (Histone H3A)-with a total sequence length of about 1.7 Kb were sequenced from 34 *Metacrangonyx longipes *specimens and the outgroups *Metacrangonyx ilvanus, M. remyi *and *M. sp *(details on sampling localities appear in Additional file 1, Figure 1 and in the Methods section). These mitochondrial sequences were assumed not to correspond to nuclear pseudogenes, as the mtDNA protein-coding genes considered did not include stop codons or frameshift mutations and no double peaks appeared in the corresponding chromatograms. Moreover, the separate analyses of each marker gave essentially congruent tree topologies, with Partition Bremer Support (PBS) positive values for most of the tree nodes (not shown). Few nodes with low support showed PBS values close to zero, suggesting that their low phylogenetic signal is not due to incongruence among markers. Most of the variation is contained in the mitochondrial genes: *cox1*, *cob *and *rrn*L had 120, 78 and 43 parsimony informative positions, respectively. Histone H3A sequences rendered five haplotypes only, with six parsimony informative sites and two fixed substitutions in *M. longipes *with respect to the outgroup species.

**Figure 1:**
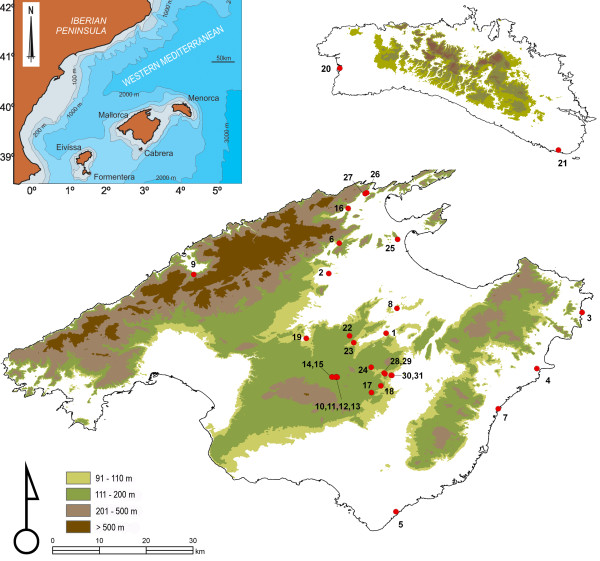
**Map of the Balearic Islands**. Sketch map of the Balearic Archipelago (W Mediterranean) and of Mallorca and Menorca islands, showing the current relief and location of sampling sites. Contour lines of +90 and +110 m roughly outline palaeogeography during the main Plio-quaternary sea-level transgressive phases, assuming little or no geological uplift or subsidence.

### Phylogenetic analyses and genetic distances

**Figure 2:**
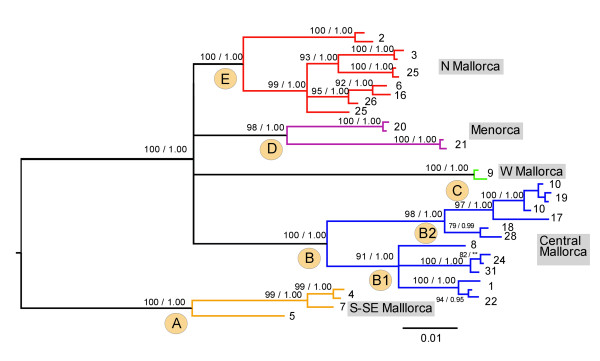
**Bayesian inference tree**. Bayesian phylogenetic tree of *Metacrangonyx longipes *based on the combined mitochondrial-nuclear data set. Values above nodes denote bootstrap values > 85% in maximum likelihood analyses (first number) and posterior probability values > 0.95 (second number).

Bayesian and maximum likelihood (ML) analyses of the combined mitochondrial and nuclear data set yielded a similar topology, in which five divergent monophyletic lineages of *M. longipes *not showing geographical overlap were clearly recognized (see Figure 1 for a map of Mallorca and Menorca and the corresponding sampling sites, and Figure 2 for the Bayesian tree). A clade comprising three anchialine caves from the S and SE of Mallorca (clade A; localities 4, 5 and 7) was highly supported and recovered as sister to the remaining clades after rooting the tree with congeneric species. The remaining populations formed four highly supported clades, although the evolutionary relationships among them remain unresolved: clade D (Menorcan, corresponding to anchialine caves 20 and 21); two divergent Mallorcan clades, one located on the west side (clade C, corresponding to freshwater cave 9) and the other on the north side of the island (clade E; localities 2, 3, 6, 16, 25 and 26, corresponding to both anchialine caves and freshwater wells); and clade B, comprising wells located far inland in the Mallorcan central area. The latter cluster was, in turn, subdivided into two genetic groups (clade B1: localities 1, 8, 22, 24, 31; and clade B2: localities 10, 17, 18, 19 and 28) showing an approximate NE-SW geographical segregation. A more comprehensive data set comprising the *cox1 *gene fragment from 162 specimens was used in population analyses (Table 1). Bayesian and ML analyses performed on the *cox1 *data set resulted in phylogenetic trees that were compatible with those derived from the combined analyses mentioned above; however, ambiguous or non-supported relationships persisted among clades, such as the position of the Menorcan populations with respect to their Mallorcan counterparts. In addition, the relationship among Mallorcan clades E and C was only weakly supported (Additional file 2). Parsimonious reconstructions of habitat type based on the *cox1 *or the total evidence mtDNA phylogenetic analysis showed at least three transitions to fresh inland groundwaters from anchialine brackish habitats (see Additional file 3).

**Table 1:** Summary of *cox1 *MtDNA population statistics

Sampling locality	**N° indv**.	N° Haplotypes	**Hap. Diversity **± **SD**	**Nuc. Diversity (**π **) × 10^2 ^**± **SD**
1	6	H17 (3), H18 (2), H20 (1)	0.733 ± 0.155	0.480 ± 0.334
2	4	H11 (1), H12 (2), H13 (1)	0.833 ± 0.222	0.235 ± 0.210
3	19	H2 (15), H3 (4)	0.351 ± 0.111	0.055 ± 0.063
4	4	H43 (1), H44 (1), H45 (2)	0.833 ± 0.222	0.157 ± 0.155
5	6	H40 (6)	0.000 ± 0.000	0.000 ± 0.000
6	2	H6 (1), H9 (1)	1.000 ± 0.500	0.313 ± 0.383
7	8	H41 (7), H42 (1)	0.250 ± 0.180	0.039 ± 0.056
8	12	H25 (10), H26 (2)	0.303 ± 0.148	0.047 ± 0.059
9	14	H37 (8), H38 (4) H39 (2)	0.615 ± 0.102	0.110 ± 0.099
10	3	H27 (1), H29 (1), H33 (1)	1.000 ± 0.272	0.417 ± 0.375
11	3	H27 (2), H32 (1)	0.667 ± 0.314	0.313 ± 0.295
12	1	H30 (1)	NC	NC
13	1	H49 (1)	NC	NC
14	3	H27 (1), H29 (1), H50 (1)	1.000 ± 0.272	0.313 ± 0.295
15	1	H31 (1)	NC	NC
16	1	H1 (1)	NC	NC
17	1	H34 (1)	NC	NC
18	1	H35 (1)	NC	NC
19	7	H27 (2), H28 (1), H29 (2), H48 (2)	0.857 ± 0.102	0.283 ± 0.212
20	5	H14 (4), H15 (1)	0.400 ± 0.237	0.063 ± 0.080
21	13	H16 (13)	0.000 ± 0.000	0.000 ± 0.000
22	2	H19 (2)	0.000 ± 0.000	0.000 ± 0.000
23	2	H19 (2)	0.000 ± 0.000	0.000 ± 0.000
24	5	H21 (5)	0.000 ± 0.000	0.000 ± 0.000
25	23	H4 (1), H5 (20), H8 (1), H46 (1)	0.249 ± 0.117	0.245 ± 0.169
26	4	H7 (3), H10 (1)	0.500 ± 0.265	0.391 ± 0.315
27	1	H47 (1)	NC	NC
28	6	H22 (4), H23 (1), H36 (1)	0.600 ± 0.215	1.701 ± 1.043
29	1	H22 (1)	NC	NC
30	1	H24 (1)	NC	NC
31	2	H22 (2)	0.000 ± 0.000	0.000 ± 0.000

Number of specimens of *Metacrangonyx longipes *per collection site, *cox1 *mtDNA haplotypes, haplotype and nucleotide diversities with corresponding standard deviations.

*Cox1 *uncorrected distances between collection sites ranged from a minimum of 0.5% between those located close to each other (viz., 18 and 28, only 3.5 km apart) to a maximum of 8.9% (corrected to 9.8% using a GTR model) between populations from the two islands (viz., localities 4 and 20, separated by 68 km) or between some Mallorcan populations. As deduced from the phylogenetic analyses, clade A was the most divergent (7.8-8% mean uncorrected genetic distance with respect to the remaining clades), whereas the distance between the other clades fell between 6.3-7.5%. The distance between subclades B1 and B2 averaged 5.5%.

### Population genetic structure and genetic diversity

Fifty different *cox1 *haplotypes were identified in the sampled specimens from the 31 populations analysed (EMBL accession numbers FR729731-FR729892) (Table 1). Four haplotypes were shared between neighbouring populations: haplotypes H27 and H29 in several central Mallorcan localities (Montuïri/Ruberts; stations 10, 14 and 19), whereas haplotypes H19 and H22 were present in two Sineu wells (stations 22 and 23). Table 1 summarizes the standard intra-population diversity estimated for *cox1*. Six populations included only one haplotype (h = 0), although in three of them only two specimens (the only ones collected) were analysed. In sharp contrast, three populations showed maximum diversity indices, as every individual bore a different haplotype (h = 1). The rest of populations attained low-to-moderate h values, in the range of 0.25-0.86. Diversity was much lower in the two Menorcan populations (three haplotypes per 28 individuals) compared with the Mallorcan populations (47 haplotypes per 144 individuals). Nucleotide diversity (mean number of pair-wise differences π) were low at most locations (π < 0.5%); population 28 alone exhibited a π value > 1% because of the presence of an individual bearing a divergent haplotype. Neutrality tests were non-significant in all cases, with the exception of populations 7 (Fu's Fs = -0.182, *P *< 0.05) and 8 (Ramos-Onsins and Rozas R_2_ = 0.51, *P <*0.01).

The Mantel test revealed a moderately significant correlation (*r *= 0.467, *P *< 0.001) between genetic and geographic distances. In addition, *cox1 *pair-wise population F*_ST _*values were generally high (F*_ST _*= 0.5-0.99) and highly significant, with the exception of between neighbouring populations (viz., 19 vs. 10-15, 24 vs. 28). Accordingly, SAMOVA showed that the optimum number of population groups necessary to maximize F*_CT _*values was *K *= 19-20 (F*_CT _*= 0.961-0.963), whereas the corresponding F*_SC _*values were the lowest in the *K *series tested, as expected [24]. This structure grouped wells 10-15 and 19 into a single population, whereas wells at Campanet (6) and Pollença (16) on one side, and wells at Vilafranca (28-31) and Cala Sant Vicenç (26, 27) on the other, formed three different populations. Most sites harbouring the same population were less than 2 km apart, with the exception of sites 10 and 19, which were separated by about 10 km. The remaining sites each represented a single, distinct population group, suggesting the occurrence of a high population subdivision with gene flow occurring exclusively among wells located less than 10 km apart. The occurrence of an alternative geographical structuring that yielded higher *K *values was explored using AMOVA, as this method allows recognition of population groups with any number of *K*. This analysis yielded a similar geographical setting, but identified wells 16, 19 and 26 as different populations; AMOVA showed that F*_CT _*values reached a plateau at 0.96 at *K *= 19-27, with the highest value attained at *K *= 23.

### Estimation of coalescence time

**Figure 3:**
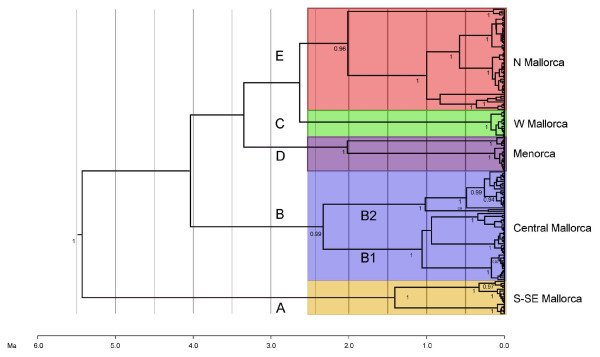
**Chronogram based on mtDNA tree**. Bayesian ultrametric tree of *Metacrangonyx longipes *obtained using an uncorrelated log-normal relaxed clock assuming a coalescent model with constant population size. Dating of major clades was performed assuming a substitution rate fixed at 2.3% pair-wise divergence per million years.

The coalescence of the mitochondrial sequences of *M. longipes *was estimated via Bayesian analyses using the *cox1 *population data set and implementing a relaxed molecular clock with a substitution rate fixed at 0.0115 per year per lineage [25], or the range 0.007-0.013 estimated elsewhere for crustaceans [26]. Tree root ages fell between 5.4 and 6.2 Ma depending on the assumed rate, while other node ages were remarkably similar in both instances, although the crustacean mitochondrial rate range rendered slightly older estimates and broader confidence intervals (Table 2 and Figure 3). Estimations using the Yule model on the combined mitochondrial data set and a standard 2.3% rate fell also in the same range (Table 2). Based on the coalescent model, divergence of the Mallorcan clade B -comprising localities from the central area of the island- can be traced back at 2.3-2.7 Ma, whereas that of clade E - occupying the N and NE of the island- seems to have occurred at 2.0-2.4 Ma. In both cases, 95% highest posterior densities (HPDs) fell within the range 3.7-1.2 Ma (Figure 3 and Table 2). Seemingly, the node corresponding to clade D (Menorca) was dated at 2.1-2.3 Ma, whereas that of clade A (comprising S and SE Mallorcan sites) was dated at 1.4-1.6 Ma (95% HPD, 3.5-0.6 Ma in both cases). Nodes corresponding to the two Mallorcan sister subclades B1 and B2 were dated at 1.1-1.2 and 1.0-1.3 Ma, respectively (95% HPD, 1.8-0.6 Ma). In contrast, the coalescence of monophyletic sequences from particular Mallorcan caves or wells was much more recent, with estimates falling within 0.1-0.2 Ma.

**Table 2:** Estimation of coalescence times

Clade	Coalescence Model *Cox1* Arthropod fixed 2.3%	Coalescence Model *Cox1* Crustacean 1.4-2.6%	Yule Model Mit. Combined Arthropod fixed 2.3%
Node A	1.37 (0.64-2.18)	1.57 (0.76-2.48)	1.83 (1.24-2.48)
Node B	2.33 (1.44-3.19)	2.66 (1.68-3.70)	2.31 (1.78-2.83)
Node C	0.17 (0.05-0.32)	0.19 (0.05-0.36)	0.11 (0.02-0.22)
Node D	2.07 (1.04-3.08)	2.35 (1.14-3.49)	2.11 (1.47-2.77)
Node E	2.04 (1.24-2.83)	2.36 (1.46-3.27)	2.26 (1.68-2.88)
Node B1	1.14 (0.68-1.62)	1.30 (0.76-1.84)	1.17 (0.83-1.54)
Node B2	1.05 (0.59-1.56)	1.21 (0.68-1.22)	1.07 (0.74-1.41)
Tree root	5.38 (3.45-7.46)	6.22 (4.10-8.70)	5.83 (4.46-7.09)

Age of the major clades shown in Figure 3, estimated using a Bayesian non-correlated relaxed molecular clock assuming a Yule tree prior, or a coalescent constant population size model and alternative calibration rates. Mean and 95% HPD values are given in million years.

## Discussion

The thalassoid condition of *Metacrangonyx longipes *is supported in our study as we can deduce at least three independent episodes of colonization of fresh inland groundwaters from primary anchialine, brackish water ancestors. *M. longipes *populations appeared split into five deep genetic lineages devoid of any relevant morphological differentiation. Gene flow between populations did not exceed 10 km and was frequently limited to occur in a radius of less than 2 km. Therefore, our results do not support an active colonization of fresh inland groundwater habitats by expansive crevicular/interstitial marine littoral ancestors (although past episodes of dispersal during favourable conditions can not be ruled out completely) [17-19]. If that was the case, we should have found evidence of substantial connectivity between the populations of *M. longipes *established far inland in completely fresh waters and those of the coastal anchialine medium, and among anchialine population themselves.

**Figure 4:**
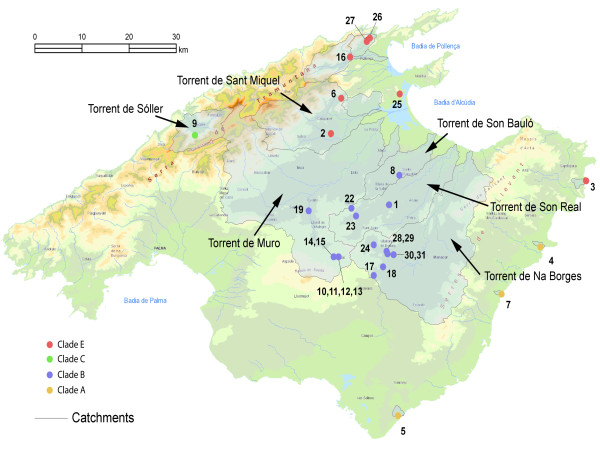
**Major Mallorcan clades and hydrographic catchments**. Map of Mallorca showing the correspondence between hydrographic catchments and distribution of major clades recognized based on mtDNA phylogenetic information. The map of Menorcan hydrographic catchments is not shown as the two sampling sites are from anchialine caves.

Some of the *M. longipes *clades were linked to particular or neighbouring hydrographic catchments and were found nowhere else (Figure 4). Thus, clade C was found exclusively at the Torrent de Sóller catchment, whereas clade B2 was restricted to the head-waters of Torrent de Muro (localities 10-15 and 19) and to some vicine stations at the Torrent de Na Borges catchment (localities 17-18 and 28). Likewise, clade B1 (localities 1, 8, 22-24 and 28-31) was found only at the head-waters of three different catchments: Torrent de Na Borges, Son Bauló and Son Real (Figure 4); nevertheless, these three torrents became recurrently confluent and formed a single palaeodrainage system in past glacial periods with lower sea-level, when the shallow shelf between Mallorca and Menorca was completely exposed sub-aerially (see below). These results suggest that quartering within and displacement along the hyporheic medium associated with these water-courses played a role in structuring the populations of the species. Even limited dispersal across the watershed of adjacent catchments seems possible, as shown above: the plains where the watershed between Torrent de Muro and Torrent de Na Borges is located harbours small, shallow perched aquifers that probably form a continuum in winter, when the area is soaked and attracts important numbers of waders and other waterbirds (D. J., personal observation).

In a study on hyalid and crangonyctoid stygobiont amphipods from W Australian calcrete aquifers, Cooper *et al*. [5] showed that the major mitochondrial *cox1 *lineages were restricted to a single isolated calcrete, whereas most of the genetic variation occurred between calcretes. Although some populations from neighbouring calcretes placed in the same palaeodrainage channel are genetically similar (suggesting the occurrence of gene flow in the past), populations do not appear necessarily clustered according to palaeodrainage channel. This pattern could result from the occurrence of gene flow or range expansion between palaeodrainages in the past, before populations became isolated in particular calcretes. The ulterior isolation of populations could be associated with a major period of aridification that affected the region between 10 and 4 Ma [5].

Major cladogenetic events in *M. longipes *can be related to the succession of past sea-level changes in the Mediterranean (with the caveat of the limitations and errors associated with molecular-clock estimations). During the Tortonian (11.3 Ma), Mallorca and Menorca were invaded by an epicontinental sea that reduced the former to a cluster of small islands roughly corresponding to its current uplands, whereas the southern half of Menorca was probably completely submerged (see Figure 1) [27]. We assume that a single *M. longipes *population was then distributed along the entire continental shelf of the archipelago. This ancestral population overcame the phase of deposition of evaporites of the so-called "Messinian Salinity Crisis", which was dated precisely at 5.96-5.59 Ma [28] and was coeval with a generalized marine regression episode that could have dried up the Mediterranean completely at that epoch. This mega-regression was probably the ultimate cause of the split of the species into two major lineages: the former clade A, corresponding to the population that remained associated and followed the receded sea coastline towards the SE; and the remaining clades, which were presumably derived from the portion of the population that followed the receded coastline towards the N (see Figure 1). The age of the most recent common ancestor of clade A and its sister group (the remaining populations) has been estimated in our analyses at ca. 5.4-6.2 Ma using a relaxed molecular clock based on *cox1 *sequences and a coalescent model.

Sometime between 4.2 and 2.7 Ma (upper-middle Pliocene; Figure 3), the populations from Menorca (node D), the Mallorcan central zone (node B), N Mallorca (node E) and W Mallorca (node C) became separated. The corresponding cladogenetic events might be linked to a single major marine transgression-regression cycle, such as that triggered by the upper Pliocene refulfilment of the depressed basins in the W Mediterranean area. The upper Pliocene transgression, which took place immediately after the Salinity Crisis, probably reached ca. +100 m above the current sea level in the Balearic area [29]. This might have enabled the species to reach the current central zone of Mallorca (Figure 1). More recently, our phylogeny shows that at the beginning of the late Pliocene, clades B, D and E experienced further splits that were followed by a differentiation of populations, with major secondary bifurcations occurring between 2.0 and 0.5 Ma. The uncertainties and large stochastic errors associated with the molecular clock estimations preclude the correlation of the tree node ages with the datings of particular geological and climate transitional episodes. However, it is remarkable that the obtained tree topology is in agreement with the documented chronological succession of changes in the late Pliocene to mid-Pleistocene sea-level record in the North Hemisphere [30-32]. We suggest that recent cladogenetic events in *M. longipes *can be linked to two major cooling events roughly dated back at 2.5 to 3 and 1.2 to 0.85 Ma, respectively.

## Conclusions

Our data suggest that marine transgression-regression cycles (eustatic changes) may have induced the repeated range expansion, contraction and fragmentation of populations of *M. longipes*, which appears currently split into several isolated and genetically divergent lineages adapted to a broad spectrum of salinity conditions. This scenario could explain the difficulty in resolving the phylogenetic relationships among different lineages of this amphipod, regardless of the method or sequence data set used: the rapid isolation and almost synchronous diversification of peripheral populations of the same ancestor in inland aquifers may have led to this situation. This hypothesis has been proposed to account for the distribution of particular anchialine and fresh groundwater taxa at various taxonomic levels and at larger geographical scales [33]. Our study stressed the importance of changes in sea level as a cause of deep intra-specific genetic divergence in thalassoid subterranean amphipods, a pattern that was apparently not accompanied by remarkable morphological differentiation [32].

## Methods

### Sampling

One hundred and sixty-two specimens of *M. longipes *were collected from seven anchialine and one freshwater cave, and from 23 freshwater wells spanning the entire geographic range of the species (Figure 2), using a modified Cvetkov net [34] and hand-held plankton nets. Individuals were preserved in 95% ethanol in the field and conserved at -20°C for subsequent molecular analyses. The sampling locations (with their geographical coordinates) and the number of individuals analysed for three mitochondrial and one nuclear marker are reported in Additional file 1. Three congeneric species were used as out-groups: the Moroccan *Metacrangonyx *sp. and *M. remyi *Balazuc & Ruffo, 1953 were collected in a well at Tamri (the coast of Agadir) and at the type locality located 1280 m above sea-level in the High Atlas, respectively. *M. ilvanus *Stoch, 1997 was collected in a well at Elba Island (Italy).

### Sequencing

Genomic DNA was isolated from whole specimens using the DNeasy Tissue Kit (Qiagen, Hilden, Germany), according to the manufacturer's recommendations. PCR was used to amplify a fragment of ~650 bp of the mitochondrial *cox1 *gene using the primers described in [35] or, in some cases, using the specific primers metacoxF2 (5'- GAACTTAGATACCCWGGTAATTTGATYGG-3') and metacoxR2 (5'- TCAGTTAATAAYATAGTAATAGCYCC-3'). Fragments of three other genes were also amplified in a subset of 34 individuals: 400 bp of the 16S rRNA (*rrn*L) gene were amplified using the specific primers 16SmetaF (5'- RGTATTTTGACCGTGCTAAGG-3') and 16SmetaR (5'- TGTAAAAATTAAARGTTGAACAAAC-3'), 360 bp of the cytochrome b (*cob*) gene were amplified using the primers described in [36], and 325 bp of the nuclear gene Histone H3A were amplified using the primers from [37]. EMBL accession numbers for the *M. longipes *individuals and outgroup species for *rrn*L, *cob *and Histone H3A are FR846024-FR846060, FR846061-FR846096 and FR846097-FR846133, respectively.

PCR was performed on a PTC-100 thermocycler (MJ Research) using a reaction volume of 25 μl and amplification conditions consisted of one cycle at 94°C for 2 min and 40 cycles of 94°C for 30 s, 47-55°C for 30 s and 72°C for 1 min, followed by a final incubation step at 72°C for 10 min. Amplified products were purified with Invitek columns (Invitek GMBH, Berlin, Germany), according to the manufacturer's instructions. The fragments were sequenced in both directions using the ABI Prism BigDye Terminator Cycle Sequencing Ready Reaction kit v. 2.0 and electrophoresed and detected on an ABI 3100 automated sequencer (Applied Biosystems, Foster City, CA, USA). Alignments were performed using MAFFT http://www.ebi.ac.uk/Tools/mafft/index.html, with default parameters.

### Phylogenetic analyses

Partition Bremer Support values were estimated using TreeRot v. 3 [38] and PAUP 4.0b10 [39]. Phylogenetic Bayesian analyses were conducted using MrBayes v. 3.1.2 [40]. We selected the model that fit the data best for each partition in the jModelTest [41] using the Bayesian information criterion. Models were tested for each of the three codon positions. The HKY+I model was selected for the first and second positions, and the GTR+G model for the third position in the case of the mitochondrial-protein-coding genes, whereas the HKY+I and F81+I models were used for *rrn*L and Histone H3A, respectively. Competing partition strategies were compared using Bayesian Information Criterion [42]. In the combined mitochondrial and nuclear data set, four partitions were favoured (first + second codon positions of *cox1 *and *cob*, third codon positions of *cox1 *and *cob, rrn*L and Histone H3A as separate partitions), whereas two partitions were selected in the case of *cox1*-only data sets (first + second vs third codon positions). Two independent runs were performed for each Bayesian search with default prior values, random trees and three heated and one cold Markov chains running for five million generations and sampled at intervals of 1000 generations. All parameters were unlinked and rates were allowed to vary freely over partitions. The burn-in and convergence of runs were assessed by examining the plot of generations against likelihood scores using the *sump *command in MrBayes. The convergence of all parameters in the two independent runs was also assessed using the Tracer program, v. 1.4 [43]. Trees resulting from the two independent runs (once burn-in samples were discarded) were combined in a single majority consensus topology using the *sumt *command in MrBayes, and the frequencies of the nodes in a majority rule tree were taken as *a posteriori *probabilities [40]. Maximum likelihood analyses using the above-mentioned partition schemes were performed using RAxML v. 7.0.4 implementing a fast bootstrapping algorithm [44]. Finally, we used Mesquite v. 2.74 [45] to reconstruct the *M. longipes *habitat character state at ancestral nodes (inland fresh vs. brackish groundwaters) using parsimony. In this analysis, we used the *cox1 *phylogenetic tree (as it represents a full population sampling) and the observed habitat distribution among populations to minimize the number of steps of habitat change.

### Population analyses

A Mantel test was performed on genetic (*cox1*) and geographic distances of populations using the ZT program [46], to check for the occurrence of isolation by distance. Population diversity indices for the *cox1 *data set, such as number of haplotypes, haplotype and nucleotide diversity, and pair-wise F*_ST _*distances and their significance based on 10,000 permutations were obtained using ARLEQUIN v. 3.01 [47] Populations represented by only one sequenced individual were excluded from the analyses. SAMOVA v. 1.0 [24] was used to identify geographical groupings that maximized genetic variance between groups of populations (F*_SC_*). The method calculates F statistics (F*_SC_*, F*_ST _*and F*_CT_*) using AMOVA [48] and identifies the optimum number of population groups for a set of sampled populations given a geographic distribution. We used 100 simulated annealing processes for each value of *K *from *K *= 2 to *K *= 20. Neutrality tests were performed for individual populations calculating Fu's F*_S _*[49] and the parameter R*_2 _*[50] using ARLEQUIN v. 3.01 and DnaSP v. 5.10.1 [51], respectively, with the latter assuming no recombination and 10,000 replicates. Simulations have shown that R*_2 _*and F*_S _*are better at detecting population growth compared with other tests, the former being superior for small sample sizes [50].

### Estimation of divergence time

Two different strategies were explored to estimate population divergence times. First we enforced the standard mitochondrial arthropod rate fixed at 2.3% pair-wise divergence per million years (0.0115 substitutions per year and lineage [25], and secondly we implemented a mitochondrial rate range of 1.4 to 2.6% substitutions per million years, that was previously estimated for marine decapods and has been frequently applied to other crustaceans [6,26,52]. In both approaches, the *cox1 *data set of the 162 sampled individuals was used applying an uncorrelated log-normal clock, assuming a coalescent model with constant population size as the best model fitting the data. BEAST [53] analyses were run starting from a random tree and using the models and partitions described for the MrBayes analyses. The remaining parameters (nucleotide frequencies and substitution model across partitions) and the rate-heterogeneity models were unlinked and estimated from the data. The search was set to 20 million generations, sampling every 1000 generations. The prior for the crustacean mitochondrial range in clock rate was implemented as a normal distribution with a mean of 0.01 substitutions per year per lineage, with maximum and minimum values of 0.013 and 0.007, respectively. The outputs of two independent runs were analysed using Tracer v. 1.4 after discarding the first 2 million generations. In another analysis, a reduced data set comprising the three combined mitochondrial genes from 34 individuals representing the major lineages was used and applied the fixed standard arthropod mitochondrial clock mentioned above but assuming a Yule model.

## Authors' contributions

MMBR performed the laboratory work. MMBR, CJ and JP carried out the molecular genetic analyses and participated in sampling. CJ drafted the manuscript. JJF participated in geological analyses. DJ participated in sampling and produced the last version of the manuscript with CJ. DJ, CJ and JP conceived the study. All authors read and approved the final manuscript.

## Supplementary Material

Additional file 1 **List of sampling sites**. Population labels, sampling sites, island, geographical position and number of specimens analysed for three mtDNA and one nuclear marker of *Metacrangonyx longipes*. Click here for file

Additional file 2 **Bayesian *cox1 *mtDNA tree**. Bayesian phylogenetic tree of *Metacrangonyx longipes *based on the *cox1 *mitochondrial data set. Values above nodes correspond to bootstrap values > 85% in maximum likelihood analyses (first number) and to posterior probability values > 0.95 (second number). Click here for file

Additional file 3 **Ancestral habitat tracing on the Bayesian *cox1 *mtDNA tree**. Parsimonious reconstruction of *M. longipes *habitat at ancestral nodes. Inland fresh groundwater and brackish groundwater populations are indicated in blue and yellow, respectively. Click here for file
