# Supplementary material for: Islands beneath islands: phylogeography of a groundwater amphipod crustacean in the Balearic archipelago
Source: BMC Evol Biol. 2011 Jul 26;11:221. doi: 10.1186/1471-2148-11-221 (PMC3161010; doi:10.1186/1471-2148-11-221)
Supplement: Additional file 1 — List of sampling sites. Population labels, sampling sites, island, geographical position and number of specimens analysed for three mtDNA and one nuclear marker of Metacrangonyx longipes. [file 1471-2148-11-221-S1.DOC]

| **Label** | **Locality** | **Island** | **Latitude** | **Longitude** | **Nº individuals sequenced for**  **cox1, *cob, rrnL and* His H3A** |
| --- | --- | --- | --- | --- | --- |
| 1 | Ariany (well) | Mallorca | 39º 38' 38.11" | 03º 04' 51.39" | 2 |
| 2 | Búger (well) | Mallorca | 39º 45' 30.92" | 02º 58' 14.93" | 2 |
| 3 | Capdepera (cave, Cova de na Barxa) | Mallorca | 39º 41' 01.98" | 03º 27' 26.70" | 2 |
| 4 | Sant Llorenç des Cardassar (cave, Cova de S'Abisament) | Mallorca | 39º 34' 34.20" | 03º 22' 13.98" | 2 |
| 5 | Santanyí (cave, Cova des Dracs des Rafal des Porcs) | Mallorca | 39º 18' 04.77" | 03º 05' 59.31" | 1 |
| 6 | Campanet (well) | Mallorca | 39º 49' 00.70" | 02º 59' 27.70" | 1 |
| 7 | Manacor (cave, Cova de Cala Varques) | Mallorca | 39º 29' 56.07" | 03º 17' 46.87" | 1 |
| 8 | Santa Margalida (well, Pou d'Hero) | Mallorca | 39º 41' 30.35" | 03º 06' 06.96" | 1 |
| 9 | Sóller (cave, Cova dels Estudiants) | Mallorca | 39º 45’ 24.19" | 02º 42’ 41.92" | 2 |
| 10 | Montuïri (well A) | Mallorca | 39º 33' 36.66" | 02º 59' 06.00" | 2 |
| 11 | Montuïri (well B) | Mallorca | 39º 33' 34.38" | 02º 59' 03.24" | -- |
| 12 | Montuïri (well C) | Mallorca | 39º 33' 35.64" | 02º 59' 09.96" | -- |
| 13 | Montuïri (well D) | Mallorca | 39º 33' 34.02" | 02º 59' 13.08" | -- |
| 14 | Montuïri (well E) | Mallorca | 39º 33' 35.52" | 02º 58' 36.90" | -- |
| 15 | Montuïri (well F) | Mallorca | 39º 33' 34.56" | 02º 58' 39.12" | -- |
| 16 | Pollença (well, Pou Ca'n Colet) | Mallorca | 39º 53' 00.86" | 03º 00' 29.22" | 1 |
| 17 | Porreres (well A) | Mallorca | 39º 31' 48.48" | 03º 03' 09.90" | 1 |
| 18 | Porreres (well B, Pou de Son Gall) | Mallorca | 39º 32' 34.44" | 03º 04' 15.60" | 1 |
| 19 | Ruberts (well) | Mallorca | 39º 38' 03.06" | 02º 55' 39.57" | 2 |
| 20 | Ciutadella (cave, Cova de S'Aigua) | Menorca | 39º 58' 21.48" | 03º 50' 08.35" | 2 |
| 21 | St. Lluís (cave, Cova de Sa Figuera) | Menorca | 39º 48' 59.40" | 04º 15' 20.56" | 2 |
| 22 | Sineu (well A) | Mallorca | 39º 38' 20.34" | 03º 00' 39.00" | 1 |
| 23 | Sineu (well B) | Mallorca | 39º 37' 32.76" | 03º 01' 07.56" | -- |
| 24 | Sant Joan (well) | Mallorca | 39º 34' 42.99" | 03º 03' 08.63" | 2 |
| 25 | Alcúdia, (cave, Cova de Son Sant Martí) | Mallorca | 39º 49' 26,59" | 03º 06' 11.31" | 3* |
| 26 | Pollença, Cala Sant Vicenç (well A) | Mallorca | 39º 54' 48.55" | 03º 02' 37.32" | 1 |
| 27 | Pollença, Cala Sant Vicenç (well B) | Mallorca | 39º 54' 42.72" | 03º 02' 26.79" | -- |
| 28 | Vilafranca de Bonany (well A) | Mallorca | 39º 34' 02.88" | 03º 04' 37.45" | -- |
| 29 | Vilafranca de Bonany (well B) | Mallorca | 39º 33' 57.72" | 03º 04' 43.86" | -- |
| 30 | Vilafranca de Bonany (well C) | Mallorca | 39º 33' 48.06" | 03º 05' 25.83" | -- |
| 31 | Vilafranca de Bonany (well D) | Mallorca | 39º 33' 46.86" | 03º 05' 31.49" | 1 |
|  |  |  |  |  |  |
