# Supplementary figures and images for: Islands beneath islands: phylogeography of a groundwater amphipod crustacean in the Balearic archipelago
Source: BMC Evol Biol. 2011 Jul 26;11:221. doi: 10.1186/1471-2148-11-221 (PMC3161010; doi:10.1186/1471-2148-11-221)

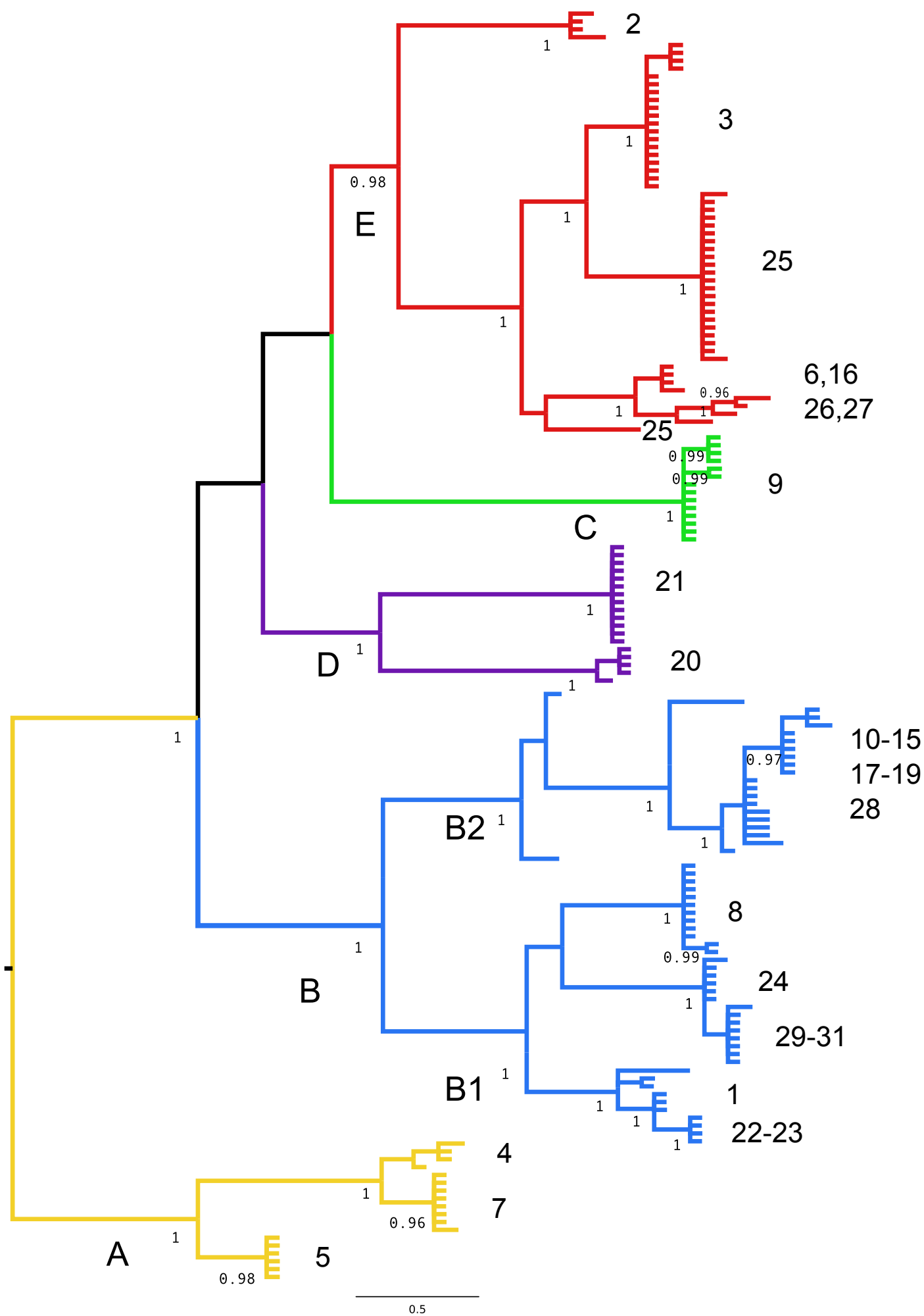

Supplement: Additional file 2 — Bayesian cox1 mtDNA tree. Bayesian phylogenetic tree of Metacrangonyx longipes based on the cox1 mitochondrial data set. Values above nodes correspond to bootstrap values > 85% in maximum likelihood analyses (first number) and to posterior probability values > 0.95 (second number). [file 1471-2148-11-221-S2.PDF]

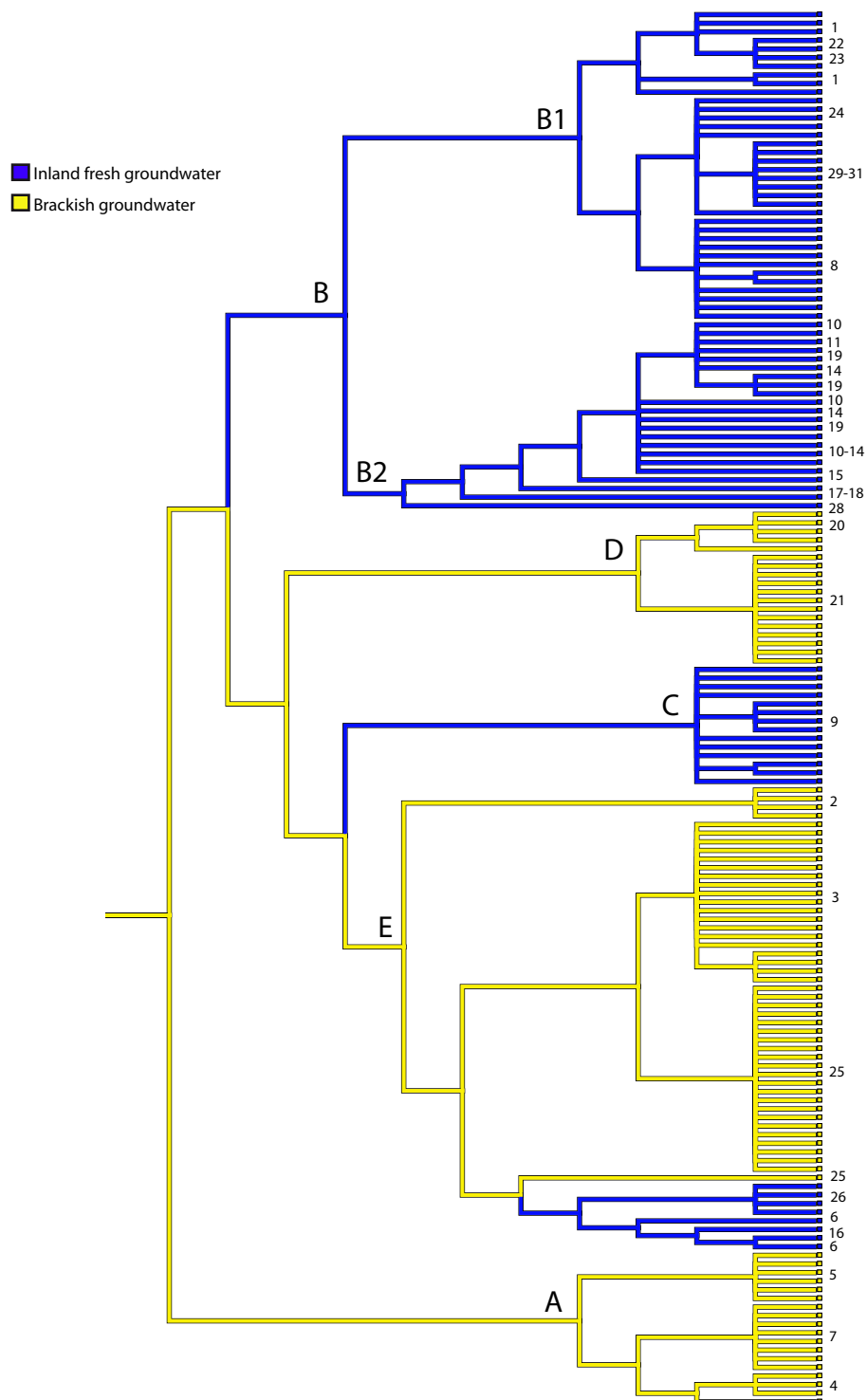

Supplement: Additional file 3 — Ancestral habitat tracing on the Bayesian cox1 mtDNA tree. Parsimonious reconstruction of M. longipes habitat at ancestral nodes. Inland fresh groundwater and brackish groundwater populations are indicated in blue and yellow, respectively. [file 1471-2148-11-221-S3.PDF]
